# Genome-wide Identification and Analysis of Splicing QTLs in Multiple Sclerosis by RNA-Seq Data

**DOI:** 10.3389/fgene.2021.769804

**Published:** 2021-11-12

**Authors:** Yijie He, Lin Huang, Yaqin Tang, Zeyuan Yang, Zhijie Han

**Affiliations:** Department of Bioinformatics, School of Basic Medicine, Chongqing Medical University, Chongqing, China

**Keywords:** multiple sclerosis, alternative splicing, RNA-seq, splicing quantitative trait loci, function analysis

## Abstract

Multiple sclerosis (MS) is an autoimmune disease characterized by inflammatory demyelinating lesions in the central nervous system. Recently, the dysregulation of alternative splicing (AS) in the brain has been found to significantly influence the progression of MS. Moreover, previous studies demonstrate that many MS-related variants in the genome act as the important regulation factors of AS events and contribute to the pathogenesis of MS. However, by far, no genome-wide research about the effect of genomic variants on AS events in MS has been reported. Here, we first implemented a strategy to obtain genomic variant genotype and AS isoform average percentage spliced-in values from RNA-seq data of 142 individuals (51 MS patients and 91 controls). Then, combing the two sets of data, we performed a *cis*-splicing quantitative trait loci (sQTLs) analysis to identify the *cis*-acting loci and the affected differential AS events in MS and further explored the characteristics of these *cis*-sQTLs. Finally, the weighted gene coexpression network and gene set enrichment analyses were used to investigate gene interaction pattern and functions of the affected AS events in MS. In total, we identified 5835 variants affecting 672 differential AS events. The *cis*-sQTLs tend to be distributed in proximity of the gene transcription initiation site, and the intronic variants of them are more capable of regulating AS events. The retained intron AS events are more susceptible to influence of genome variants, and their functions are involved in protein kinase and phosphorylation modification. In summary, these findings provide an insight into the mechanism of MS.

## Introduction

Multiple sclerosis (MS) is a serious autoimmune disease of central nervous system (CNS) and is characterized by inflammatory demyelinating lesions in the white matter (Compston and Coles, 2008). According to the most recent survey in 2020 (the *Atlas of MS* investigation), the estimated number of the people affected by MS has reached approximately 2.8 million worldwide (Walton et al., 2020). Similar to most of the complex diseases, genetic factors are the major contributors to the individual differences in MS susceptibility, and the role of genetic variants and transcriptional regulation in MS may be the key to understanding its pathogenesis (Fugger et al., 2009; Olsson et al., 2017; Yang et al., 2019).

Recently, alternative splicing (AS), a process that enables a gene to generate different transcript isoforms, has been found to have the characteristic of high complexity and play an important role in primates and human CNS (Barbosa-Morais et al., 2012; Merkin et al., 2012; GTEx Consortium, 2015; GTEx Consortium, 2020). Further, previous studies demonstrate that dysregulation of AS events in genes significantly influences the progression of many nervous system diseases, including MS. For example, the RNA helicase DDX39B, a repressor of AS of IL7R exon 6, is downregulated in MS peripheral blood mononuclear cells, and consequently, the overexpression of the soluble form of the interleukin-7 receptor alpha chain gene (sIL7R) increases MS risk (Galarza-Munoz et al., 2017). Inclusion of AS4 exon in Nrxn 1-3 is significantly increased in the prefrontal cortex of a murine MS model, and the abnormal splicing promotes the expression of IL-1β, which is an important mediator of inflammation and leading to cognitive dysfunction in MS (Marchese et al., 2021). The dysregulated AS of the A1β transcript results in a significantly diminished adenosine A1 receptor protein, which is an important therapeutic target in the treatment of MS in peripheral blood mononuclear cells and brain tissue of MS patients (Johnston et al., 2001).

Moreover, previous studies demonstrate that genetic variants can control the regulation of AS events by directly altering nucleotide sequences in the splice site or as splicing quantitative trait loci (sQTLs) in a genome-wide manner (Battle et al., 2014; GTEx Consortium, 2015; Takata et al., 2017; GTEx Consortium, 2020). For MS, numerous disease-related risk single nucleotide polymorphisms (SNPs) have been identified by genome-wide association studies (GWAS) (International Multiple Sclerosis Genetics Consortium et al., 2013; Sawcer et al., 2014; Patsopoulos, 2018), and a part of them as the regulation factors of AS events can contribute to the pathogenesis of MS. For instance, MS risk variants rs35476409 and rs61762387 can affect the splicing of exon 3 of the PRKCA gene, which is considered to be a functional contributor to MS predisposition (Paraboschi et al., 2014). Another MS risk SNP rs6897932 locates in the functional AS exon of IL7R. Through disrupting the exonic splicing silencer, it can increase skipping of IL7R exon 6 to produce more soluble and membrane-bound isoforms of IL7R protein (IL7Ra), which is a key factor in the immune response pathway of MS (Gregory et al., 2007). The SNP rs3130253, located within the MOG gene, has a proven genetic susceptibility to MS. The minor allele (A) of rs3130253 is associated with the increased splicing of MOG exon 2 to 3 in the oligodendrocyte cell (1.7-fold) and influences the extracellular and transmembrane domains of MOG to induce the development of MS (Jensen et al., 2010). Although these findings provide valuable insights into the direct influence of SNPs on AS events in MS, the profile and function of sQTLs throughout the genome remain poorly understood.

**FIGURE 1:**
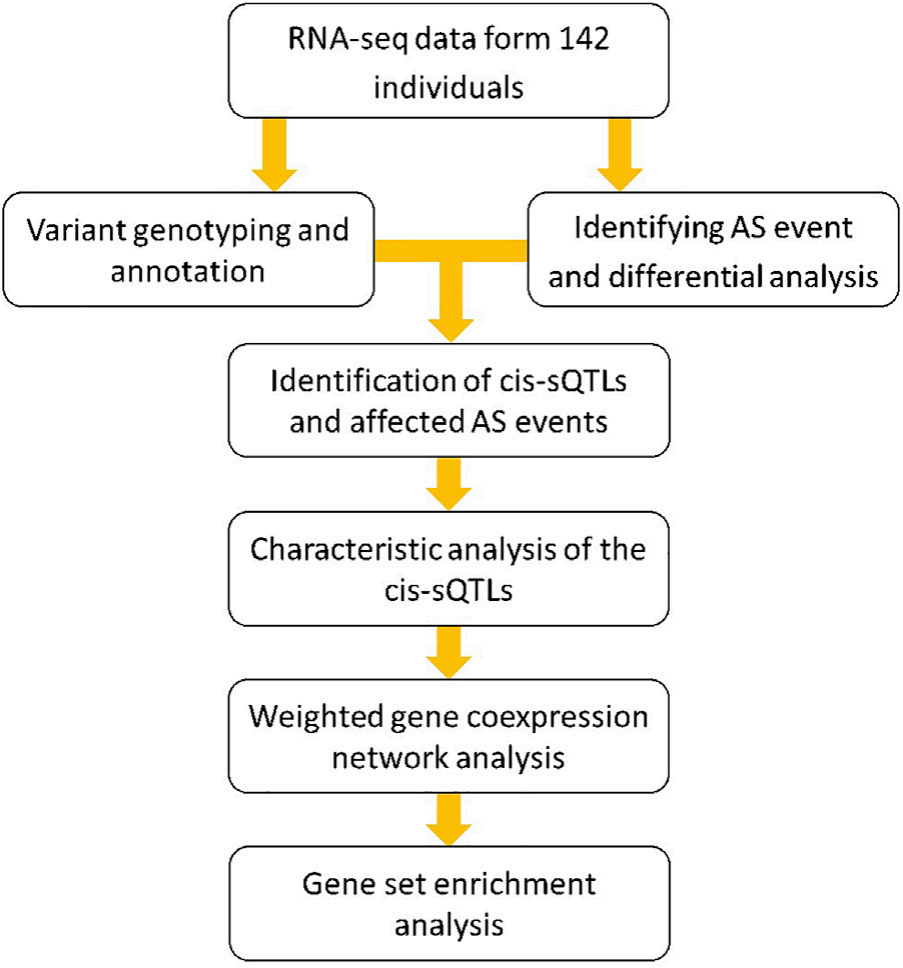
The flow chart of the study design for exploring the influence of genome variants on gene AS and their functions to pathogenesis of MS.

Our previous studies systematically describe the influence of genomic variants on gene expression in a genome-wide manner and find that this impact is more significant among the regions of long intergenic noncoding RNA for MS (Han et al., 2018; Han et al., 2020). However, by far, the genome-wide research about the effect of these genomic variants on AS events in MS has been not yet reported. To solve this problem, in this study, we used the blood RNA-seq data from 51 MS patients and 91 controls of European descent that have been previously successfully used for our expression quantitative trait loci (eQTLs) analysis (Han et al., 2020). Particularly, we first comprehensively detected the AS events on a whole-genome scale and performed a differential splicing analysis between the MS patients and healthy individuals by using the RNA-seq data. Then, based on the same data, we genotyped the large-scale genomic variants (mainly the SNPs) in the entire human genome. According to the previous studies, genotyping using RNA-seq can be effectively performed in a lower sample scale (typically tens to hundreds of individuals) and higher genetic heterogeneity and is more conducive to the discovery of functional SNPs than the traditional approaches (e.g., SNP arrays) (Wang et al., 2009; Davey et al., 2011). Next, combining the data of AS isoform average percentage spliced-in (PSI) and genomic variant genotype, we performed a sQTL analysis to identify the *cis*-acting loci and the affected AS events in MS. Further, we explored the distribution characteristics and disease specificity of these *cis*-sQTL loci. Finally, we conducted the weighted gene coexpression network analysis (WGCNA) and gene set enrichment analysis (GSEA) to investigate the interaction pattern of the AS affected genes and the functions of these genes to the pathogenesis of MS. The flow chart is shown in Figure 1.

## Materials and Methods

### Sample Collection and Preprocessing

A total of 142 individuals, including 51 MS patients and 91 age- and gender-matched healthy controls, were selected from the Utrecht Medical Center (UMCU) and VU University Medical Center (VUMC) of Netherlands. The RNA-seq data of blood samples from these individuals were used for this study (Table 1). The details are described in previous studies (Best et al., 2017; Han et al., 2020). Briefly, the mirVana miRNA isolation kit was used to extract the total RNA of these samples. The Truseq Nano DNA Sample Preparation Kit and Illumina Hiseq 2500 platform were used for library preparation and sequencing, respectively. After the RNA read quality control, these sequence data were stored in the NCBI Sequence Read Archive (SRA) database (SRP093349). We used the SRA Toolkit software to download these sequence data and converted them into FASTQ files.

**TABLE 1:** Summary of the 142 individuals studied in this work.

Individuals	Institution	Ethnicity	Sample size	Mean age (s.d.)	Male/female (%)
MS patients	VUMC	European	51	46.14 (7.54)	25.5/74.5
Healthy controls	VUMC and UMCU	European	91	46.92 (8.50)	34.1/65.9
Total			142	46.64 (8.18)	31.0/69.0

VUMC, VU University Medical Center; Amsterdam, Netherlands; UMCU, Utrecht Medical Center, Utrecht, Netherlands. This information is also described in our previous study (Han et al., 2020).

### Variant Genotyping and Annotation

The procedure of variant genotyping and annotation on a whole-genome scale using FASTQ files has been described in our previous study (Han et al., 2020). Briefly, the BWA software was first used to align the sequenced reads to the human reference genome (hg19) with its default parameter settings and generated the sequence alignment/map (SAM) files (Li and Durbin, 2009). Then, the SAMtools and BCFtools software were used with their default parameter settings to perform the format conversion of these SAM files and variant calling, respectively (Li, 2011; Li et al., 2009). The genotyped variants were stored in the VCF file. Further, based on the annotation databases, refGene (about the functional information of variants) (Pruitt et al., 2007) and snp138 of dbSNP (about the genomic position and ID of variants) (Day, 2010), we used the ANNOVAR software to annotate these genotyped variants (Yang and Wang, 2015). Finally, we preformed quality control, which is based on the sequencing quality and variant annotation. We conducted a Hardy-Weinberg equilibrium (HWE) test using the R package ‘Genetics’ (https://cran.r-project.org/web/packages/genetics/index.html). According to the findings of previous studies (Greif et al., 2011; Quinn et al., 2013), we filtered the low-quality genotyped variants if their HWE *p* value <5 × 10^−5^ or root mean square (RMS) mapping quality <10 or read depth (DP) < 10 or minor allele frequency (MAF) < 1%. Moreover, other studies suggest that only the results catalogued in dbSNP should be retained to reduce the false positives when performing the SNP calling (Chepelev et al., 2009; Cirulli et al., 2010; Liu et al., 2012; Xu et al., 2013). Therefore, we further removed genotyped variants that are not catalogued in dbSNP according to the annotation results.

### Identification and Differential Analysis of AS Events

Based on the RNA-seq data of the same samples, we used the vast-tools software to detect the AS events and calculate their PSI values on a whole-genome scale (Irimia et al., 2014). In particular, we first aligned the sequenced fragments to human reference genome (hg19) using the align tool module of vast-tools software with its default parameters to identify AS events and calculate their PSI values in each sample. Then, the results (five subfiles for each AS event) were merged using the combine tool module of vast-tools software to generate a file containing PSI of each AS event and quality control content for all samples. The quality control threshold is according to quality scores in the merged file, i.e., the mapped reads >10. Next, we used the multiple imputation method with the generalized linear model to impute missing PSI values of each AS event by the R package “mice” (Van Buuren and Groothuis-Oudshoorn, 2011) and counted the number of each type of AS events. Finally, based on the PSI values, we used the diff tool module of vast-tools software with its default parameters to perform a Bayesian inference-based differential AS analysis. The threshold of significance was set at the minimum value for absolute value of differential PSI between MS cases and controls (MV|ΔPSI|) at 0.95 confidence level greater than 10% according to the previous studies (Fagg et al., 2020; Ha et al., 2021; Hekman et al., 2021).

### Identification of *cis*-s Quantitative Trait Loci and Characteristic Analysis

Combining the PSI values of AS events and the data of the genomic variant genotype from the same samples, we performed an sQTL analysis to identify the *cis*-acting loci and the affected AS events. Particularly, according to previous studies (GTEx Consortium, 2015; GTEx Consortium, 2020), we first considered it as the cis region where the distance between variants and transcription initiation site (TSS) of AS event corresponding genes less than 1 M, and selected all the suitable variant and AS event pairs for the *cis*-sQTL analysis. The genomic locations of the variants and the TSS of AS event corresponding genes are based on the annotation files of the dbSNP (snp138) and Ensembl databases (release 75), respectively. Then, we used the genotype data of the variants in combination with the PSI values of AS events to perform the sQTL analysis by the R package “Matrix eQTL” with a linear regression model (Shabalin, 2012). The parameters age and gender were used as the covariates. The threshold of significance level was set at a false discovery rate (FDR) q value <0.05. The *p* values are corrected for multiple testing by the Benjamini–Hochberg method. Finally, we calculated the percentage of various types of the *cis*-sQTL variants and the affected AS events, respectively, and compared them with the original proportion using a two-tailed Fisher exact test (the threshold of *p* < .05). Moreover, we further explored the relationship between the abundance of the *cis*-sQTL variants and the distance of them to the nearest TSS.

### Weighted Gene Coexpression Network Analysis and Gene Set Enrichment Analysis

To explore the interaction pattern of the AS affected genes and their functions to the pathogenesis of MS, we performed the WGCNA and GSEA in turn. Particularly, we first downloaded the gene expression count data of the 51 MS patients and 91 healthy individuals from Gene Expression Omnibus (GEO) data set GSE89843 (Best et al., 2017) and carried out a standardized processing of these data using the “preprocess” function of R package “caret” (https://cran.r-project.org/web/packages/ caret/). Then, we conducted quality control to identify the outlier samples using the “hclust” function of R package “WGCNA” (Langfelder and Horvath, 2008). Further, to ensure the scale-free topology criterion of the coexpression network, we used the “pickSoftThreshold” function of R package “WGCNA” to choose the satisfactory soft threshold power β. Next, based on the satisfactory soft threshold power β, we used Pearson’s method to calculate the weighted correlation of gene pairs in an adjacency matrix and used the dynamic cut-tree algorithm to construct the hierarchical clustering dendrogram by the R package “WGCNA.” Finally, we calculated the correlation between the module membership and the importance of genes in this module to clinical traits to assess the relationship between the coexpression module and the clinical traits (including gender, age, and disease status) by the R package “WGCNA.”

We further use the genes in the modules that are significantly associated with MS disease status to perform GSEA by DAVID software (Jiao et al., 2012). The default background of DAVID, i.e., three pathway data sets (BBID, BIOCARTA, and KEGG_PATHWAY), three gene ontology data sets (GOTERM_BP_DIRECT, GOTERM_CC_DIRECT, and GOTERM_MF_DIRECT), three functional categories (COG_ONTOLOGY, UP_KEYWORDS, and UP_SEQ_FEATURE), three protein domains (INTERPRO, PIR_SUPERFAMILY, and SMART), and one disease data set (OMIM_DISEASE) for the GSEA. The threshold of significance was set at FDR q < 0.05. The other parameters were set according to the default values of the DAVID software.

## Results and Discussion

### Variant Genotyping by RNA-Seq Data

**FIGURE 2:**
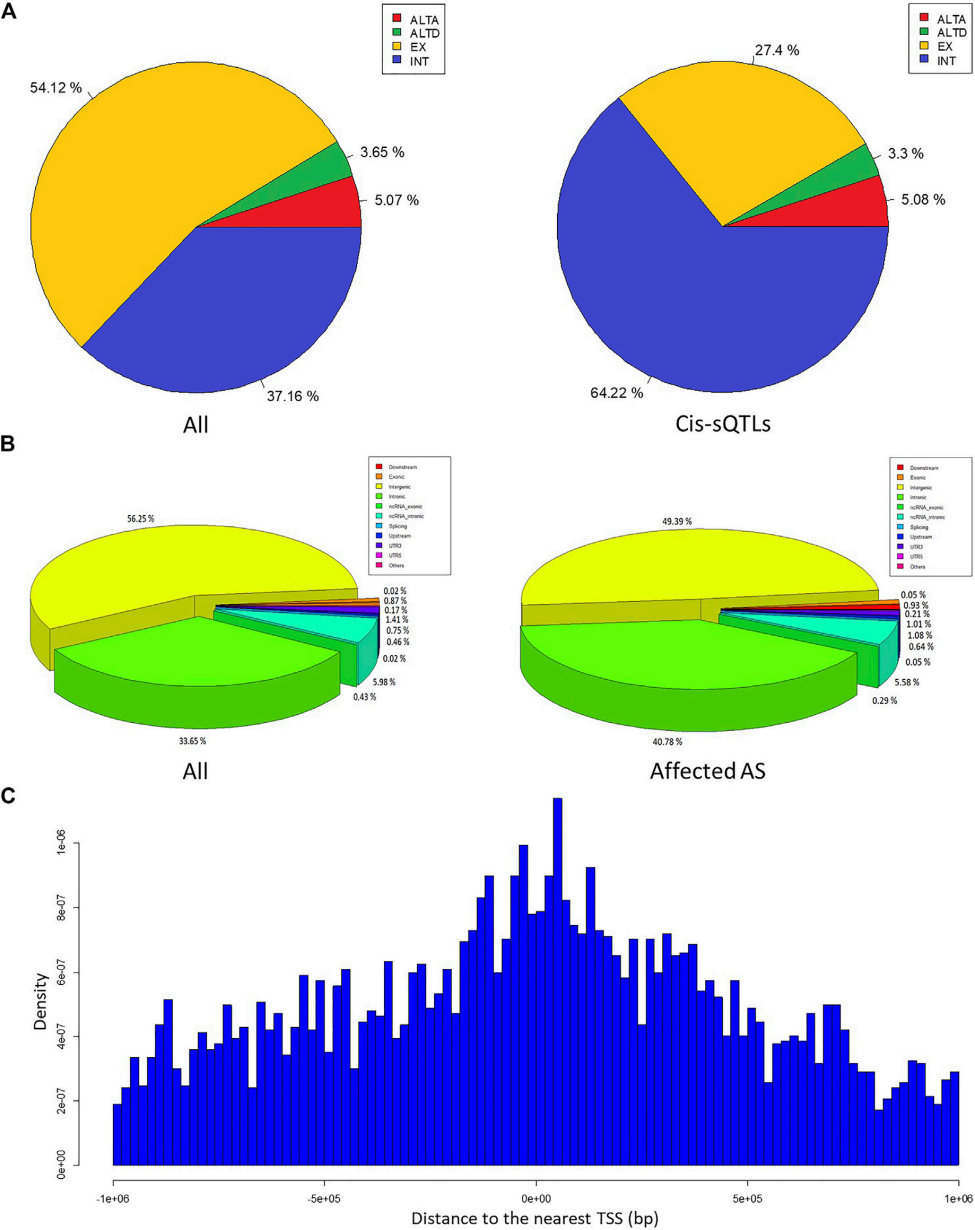
The characteristic of the *cis*-sQTL variants and the affected AS events. **(A)** The pie charts show the percentage of all variants (left) and *cis*-sQTL variants (right) annotated with each class (intergenic, intronic, exonic, ncRNA intronic, ncRNA exonic, 5′/3′-UTR, upstream/downstream, splicing site, and others), respectively. **(B)** The pie charts show the proportion in all AS events (left) and affected AS events (right) annotated with each class (EX, INT, ALTA, and ALTD), respectively. (**C**) The bar graph indicates the relationship between the abundance of the *cis*-sQTL variants and the distance of them to the nearest TSS of AS events corresponding genes.

We obtained a total of about 3.2 billion sequenced reads from the blood RNA-seq data of 51 MS patients and 91 healthy controls. Based on these RNA-seq data, we aligned the sequenced reads to human reference genome (hg19) using BWA software and used these aligned reads to call the variant genotypes by SAMtools and BCFtools software. After quality control based on DP, RMS mapping quality, MAF, HWE, and dbSNP catalog, we obtained 620,339 genotyped variants. Finally, the results of annotation using ANNOVAR software showed that a total of 600,872 genotyped SNPs and 19,467 indels are included in these genotyped variants, and approximately 56.25%, 33.65%, 0.87%, 5.98%, 0.43%, 1.58%, 1.21%, and 0.02% of them are categorized into the intergenic, intronic, exonic, ncRNA intronic, ncRNA exonic, 5′/3′-UTR, upstream/downstream, and splicing site classes, respectively. These findings reveal an uneven distribution of these variants in the genome (Figure 2A).

### Identification and Differential Analysis of Alternative Splicing Events

**FIGURE 3:**
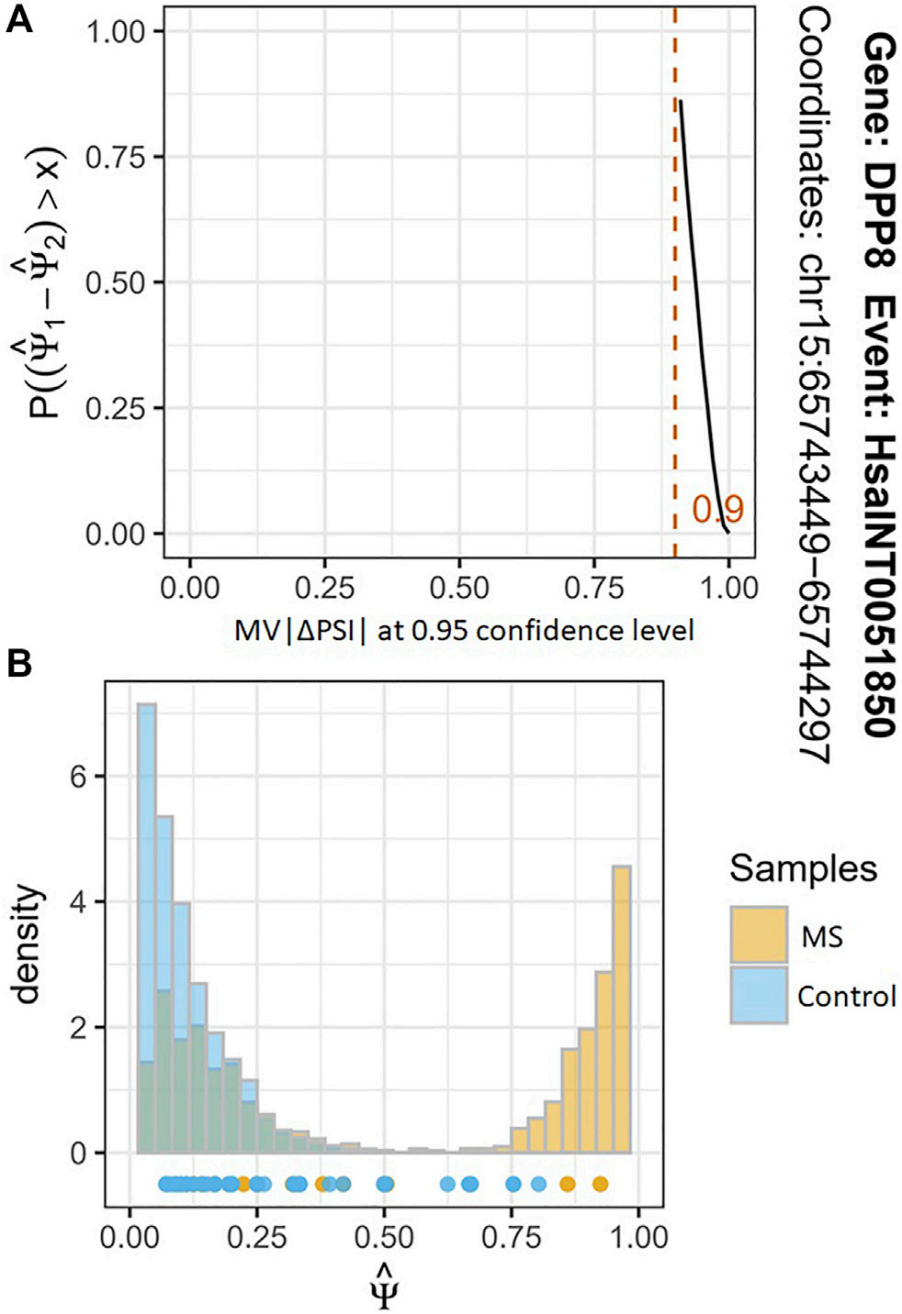
The results of differential analysis of AS event HsaINT0051850. (**A**) The *x*-axis represents MV|ΔPSI | at a 95% confidence level. The *y*-axis represents the probability of ΔPSI being greater than some magnitude value of x. The red line indicates that the maximum probability of ΔPSI of AS event HsaINT0051850 between MS cases and controls is greater than 0.90. (**B**) The histogram shows the two joint posterior distributions over PSI and the points below the histograms estimate for each replicate.

Based on the FASTQ files from the same samples, we used the corresponding tool modules of vast-tools software to identify the AS event with their PSI values and performed the differential analysis of them. After the quality control, we found a total of 2272 significant differential AS events between the MS cases and healthy individuals (MV|ΔPSI| at 0.95 confidence level ≥10%) from the more than seven million identified AS events. These differential AS events are involved in 1542 genes (Supplementary Table S1). Figure 3 shows the most significant differential AS event HsaINT0051850 of DPP8 gene (MV|ΔPSI| at 0.95 confidence level = 0.90). According to the classification criteria of vast-tools, the types of AS events contain alternative exon skipping (EX), retained intron (INT), alternative splice site acceptor choice (ALTA), and alternative splice site donor choice (ALTD). We found that approximately 54.12%, 37.16%, 5.07%, and 3.65% of these identified AS events are categorized into EX, INT, ALTA, and ALTD classes, respectively, which also revealed an uneven distribution of them (Figure 2B).

### Identification of *Cis*-s Quantitative Trait Loci and Characteristic Analysis

Combining the PSI values of AS events with the genotype data of the genomic variant in the cis region from the same samples, we used a linear regression model to perform the *cis*-sQTL analysis by R package “Matrix eQTL” with the parameters age and gender serving as covariates. In total, we identified 5835 variants affecting 672 AS events (involving 482 genes) of all these 2272 significant differential AS events with a significance level of q < 0.05. The top 30 significant results are shown in Table 2 (the full information is presented in Supplementary Table S2). Further, we found that approximately 49.39%, 40.78%, 0.93%, 5.58%, 0.29%, 1.22%, 1.72%, and 0.05% of the *cis*-sQTL variants are categorized into the intergenic, intronic, exonic, ncRNA intronic, ncRNA exonic, 5’/3′-UTR, upstream/downstream, and splicing site classes, respectively (Figure 2A), and approximately 27.40%, 64.22%, 5.08%, and 3.30% of the affected AS events are categorized into EX, INT, ALTA, and ALTD classes, respectively (Figure 2B). By the two-tailed Fisher exact test, we found that the percentage of main types both in the *cis*-sQTL variants and the affected AS events show a significant difference compared with the original proportion. Particularly, the percentage of the *cis*-sQTL intergenic variants is 49.39%, but its original proportion in all of the variants is 56.25% (odds ratio (OR) = 0.76, *p* = 1.84 × 10^−45^); the percentage of the *cis*-sQTL intronic variants is 40.78%, but its original proportion in all of the variants is only 33.65% (OR = 1.36, *p* = 1.09 × 10^−52^); the percentage of the affected EX events is 27.40%, but its original proportion in all AS events is 54.12% (OR = 0.32, *p* = 9.65 × 10^−69^); the percentage of the affected INT events is 64.22%, but its original proportion in all AS events is only 37.16% (OR = 3.03, *p* = 5.12 × 10^−70^). This reveals a specific regulation of the AS events by variants in MS. Moreover, we also found that these *cis*-sQTL variants tend to be distributed in the proximity of the TSS of AS events corresponding genes (Figure 2C).

**TABLE 2:** The top 30 significant results of the sQTL variants and the differential AS events affected by them.

SNP ID	Position	Gene	Ensembl ID	AS event	TSS	Beta	*p* Value	FDR q value
rs1950969	94236929	GOLGA5	ENSG00000066455	HsaEX0027985	93260576	34.2500	0.00E + 00	1.05E-303
rs1950970	94236975	GOLGA5	ENSG00000066455	HsaEX0027985	93260576	34.2500	0.00E + 00	1.05E-303
rs8017818	93651054	GOLGA5	ENSG00000066455	HsaEX0027985	93260576	−34.2500	0.00E + 00	1.05E-303
rs12226058	43190629	ACCSL	ENSG00000205126	HsaEX6001613	44069531	−12.7000	0.00E + 00	1.05E-303
rs12795809	43190576	ACCSL	ENSG00000205126	HsaEX6001613	44069531	12.7000	0.00E + 00	1.05E-303
rs61690000	43523415	ACCSL	ENSG00000205126	HsaEX6001613	44069531	−12.7000	0.00E + 00	1.05E-303
rs72898940	43315617	ACCSL	ENSG00000205126	HsaEX6001613	44069531	−12.7000	0.00E + 00	1.05E-303
rs74545163	43424312	ACCSL	ENSG00000205126	HsaEX6001613	44069531	−12.7000	0.00E + 00	1.05E-303
rs7931142	43189976	ACCSL	ENSG00000205126	HsaEX6001613	44069531	−12.7000	0.00E + 00	1.05E-303
rs890245	43201830	ACCSL	ENSG00000205126	HsaEX6001613	44069531	12.7000	0.00E + 00	1.05E-303
rs113384165	26738788	NSMCE1	ENSG00000169189	HsaEX6042948	27280115	−40.0000	0.00E + 00	1.05E-303
rs6498005	27270200	NSMCE1	ENSG00000169189	HsaEX6042948	27280115	−40.0000	0.00E + 00	1.05E-303
rs7187853	27267403	NSMCE1	ENSG00000169189	HsaEX6042948	27280115	−40.0000	0.00E + 00	1.05E-303
rs2976708	125398800	SNX4	ENSG00000114520	HsaEX6058167	125239041	5.1100	0.00E + 00	1.05E-303
rs543453	3139759	PIAS4	ENSG00000105229	HsaEX6091950	4007748	1.2500	0.00E + 00	1.05E-303
rs644193	3139715	PIAS4	ENSG00000105229	HsaEX6091950	4007748	1.2500	0.00E + 00	1.05E-303
rs16949296	45984949	SCRN2	ENSG00000141295	HsaEX6023334	45918699	−66.6580	3.86E-238	1.75E-233
rs11643492	2791938	SRRM2	ENSG00000167978	HsaEX6041902	2802330	52.7000	1.46E-172	6.04E-168
rs2858609	49620817	PIM3	ENSG00000198355	HsaEX6027387	50354161	22.1493	2.47E-124	9.47E-120
rs73179160	50082716	PIM3	ENSG00000198355	HsaEX6027387	50354161	−11.0746	2.47E-124	9.47E-120
rs11671147	8227499	ELAVL1	ENSG00000066044	HsaEX0022092	8070529	49.6873	2.55E-56	7.47E-52
rs62638003	7908051	ELAVL1	ENSG00000066044	HsaEX0022092	8070529	49.6873	2.55E-56	7.47E-52
rs216272	3013971	PIAS4	ENSG00000105229	HsaEX6091950	4007748	−0.5013	5.37E-50	1.53E-45
rs57414916	141780202	EIF2C2	ENSG00000123908	HsaEX6082596	141645718	−5.0980	7.11E-50	1.97E-45
rs2020857	15030752	USP9Y	ENSG00000114374	HsaEX0070061	14813160	−8.2372	1.40E-49	3.66E-45
rs138123250	105087582	CALHM2	ENSG00000138172	HsaEX6090238	105212660	−8.8666	1.27E-45	3.16E-41
rs12610435	8021331	ELAVL1	ENSG00000066044	HsaEX0022092	8070529	42.5593	5.34E-45	1.30E-40
rs192519226	48400006	XYLT2	ENSG00000015532	HsaEX6023498	48423453	−22.2205	2.90E-44	6.55E-40

### Weighted Gene Coexpression Network Analysis for Affected Alternative Splicing Events Corresponding Genes

**FIGURE 4:**
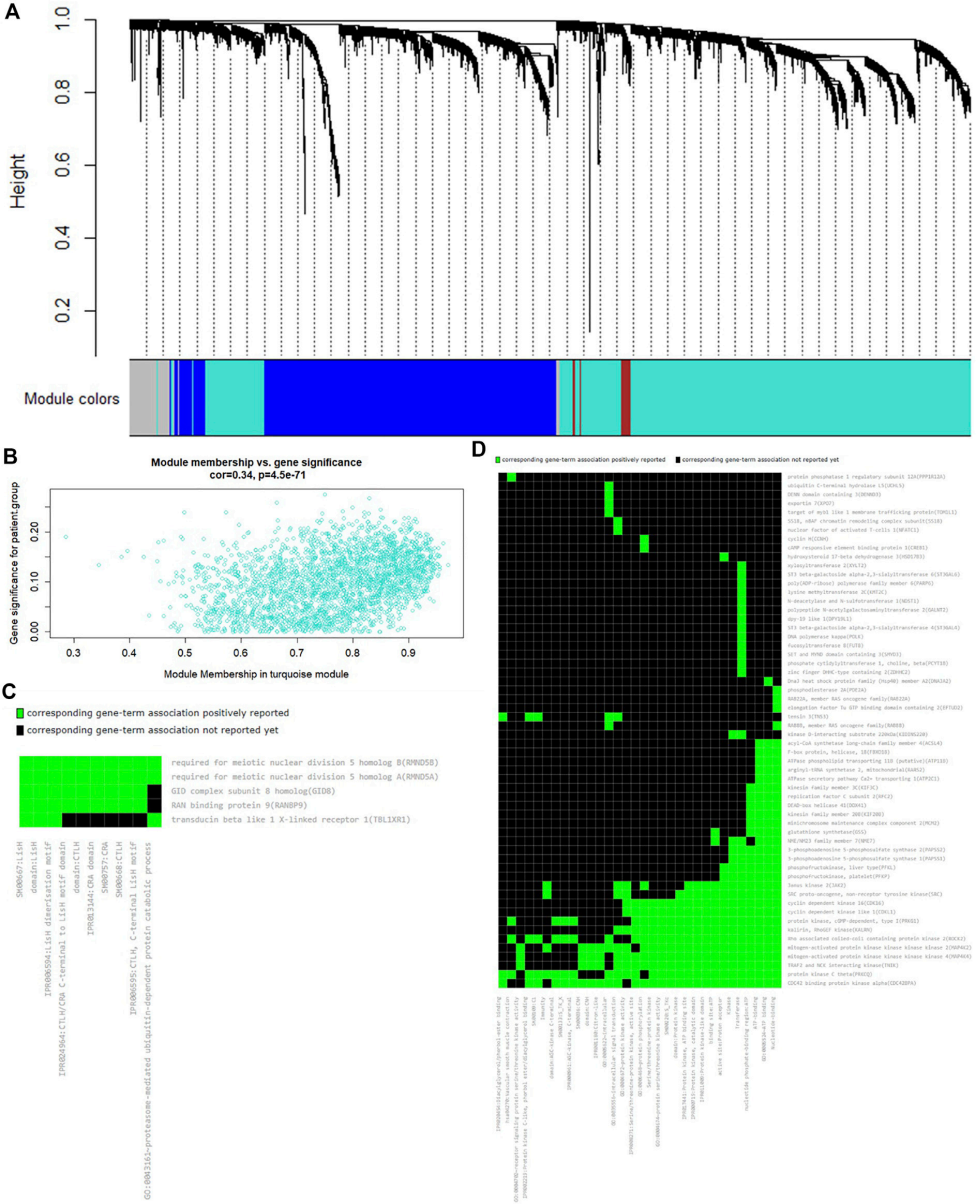
The results of WGCNA and GSEA. **(A)** The expression clustering dendrogram of all 4722 genes in the GSE89843 data set. There are four clustered modules in the hierarchical clustering dendrogram, which contain 360 of 482 affected AS events corresponding genes. These clustered modules are marked as four different colors, respectively, i.e., turquoise, blue, brown, and grey. **(B)** The correlation between the module membership and the gene significance in the turquoise module, which reveals a relatively strong correlation with the disease status (cor = 0.34 and *p* = 4.5 × 10^−71^). The gene significance is defined as the correlation between a single gene expression and sample trait (e.g., gender, age, and disease status) **(C)** The annotation cluster 1 contains 10 functionally highly similar enriched terms involved in the protein–protein interaction domain motif. (**D**) The annotation cluster 2 contains 32 functionally highly similar enriched terms involved in protein kinase and phosphorylation modification. This figure can be viewed more clearly by enlarging in the electronic version.

We performed WGCNA to explore the characteristics of the affected AS event corresponding genes in MS. According to the sample clustering results for quality control, we removed eight outlier samples (Supplementary Figure S1). Then, we found that the model fitting index R-squared reaches 0.85 for the first time, and the mean connectivity approaches zero simultaneously when the soft threshold power β equals 12 (Supplementary Figure S2). Therefore, we calculated the weighted correlation of gene pairs and constructed the coexpression network using the R package “WGCNA” with the parameter β = 12. The results show that there is a total of four modules (i.e., MEturquoise, MEblue, MEbrown, and MEgrey) in the coexpression network. The modules are defined as clusters in which the densely interconnected genes are coexpressed with each other. The unsupervised clustering analysis with a topological overlap index was used to measure the network interconnectedness. They contain a total of 4722 clustered genes according to their interconnectedness, and 360 of them belong to the affected AS event corresponding genes (Figure 4A). These AS affected genes are generally evenly distributed in the four modules according to their scale. The results of correlation analysis reveal some association of all the modules with individual gender or age (*p* < .05). Among them, however, only the turquoise module shows a relatively strong correlation with the disease status (cor = 0.34 and *p* = 4.5 × 10^−71^) (Figure 4B), which means that the interaction of the genes in the turquoise module is relevant to pathogenesis of MS. In the grey module, for example, the cor and *p* value are −.027 and .65, respectively.

### Gene Set Enrichment Analysis of Alternative Splicing Affected Genes in Multiple Sclerosis–Related Module

Based on the results of WGCNA, we used the 198 AS affected genes in the MS-related turquoise module to perform the GSEA. According to the significance threshold FDR q < 0.05, we identified a total of 30 enriched terms. The most significant of them contain the AS-related terms, e.g., alternative splicing (q = 2.0 × 10^−8^) and splicing variant (q = 1.0 × 10^−3^), which are consistent with the findings of sQTL analysis. Most of the other significant enriched terms are involved in epigenetic modification, which is the common biological process associated with the pathogenesis of MS (Supplementary Table S3). Further, we performed a functional annotation clustering analysis of the enriched terms. We identified two annotation clusters with enrichment score more than 2, which contain 10 and 32 functionally highly similar terms, respectively. Particularly, annotation cluster 1 (enrichment score = 3.78) contains the protein–protein interaction domain (e.g., LisH, CTLH, and CRA) motif-related terms, which are the basic biological properties for eukaryotes (Figure 4C). The annotation cluster 2 (enrichment score = 2.12) contains protein kinase and phosphorylation modification terms, which are significantly associated with the pathogenesis of MS (Figure 4D). For example, Feng *et al.* found that the type I interferons and the p38 MAP kinase can induce tyrosine and serine phosphorylation of STAT1 in MS patients, respectively, and the excessive phosphorylation of STAT1 can induce inflammatory cytokines and demyelination to aggravate the development of MS (Feng et al., 2002). Trinschek *et al.* found that phosphorylation of protein kinase B/c-Akt in MS autoaggressive T effector cells (Teff) is able to induce the unresponsiveness of the CD4^+^ and CD8^+^ course independent MS-Teff by stimulation of the active regulatory T cells and thereby lead to the ineffective treatment of MS (Trinschek et al., 2013). Delgado-Roche *et al.* found that ozone therapy can promote the phosphorylation of the transcriptional factor NF-E2-related factor 2 through upregulating the expression of MAP kinase CK2, which can reduce oxidative stress and pro-inflammatory cytokines in MS (Delgado-Roche et al., 2017).

## Conclusions

In this study, based on the MS RNA-seq data, we genotyped 620,339 variants and identified 2272 significant differential AS events in the same samples. Then, combing the two sets of data, we performed a *cis*-sQTL analysis and identified 5835 variants affecting 672 differential AS events in MS. Further, the results of characteristic analysis showed that the intronic variants are more capable of regulating AS events, and INT AS events are more susceptible to the influence of genome variants. Moreover, the *cis*-sQTL variants tend to be distributed in the proximity of the TSS of AS events corresponding genes. Finally, the results of WGCNA and GSEA demonstrate that the regulation of AS by genome variants are important to MS and their potential function may be involved in protein–protein interaction domain motif protein phosphorylation modification. All in all, we performed a strategy to explore the regulation of AS by genome variants in MS by RNA-seq data, and these findings will benefit the improvement of understanding MS pathogenesis.

## Data Availability

The original contributions presented in the study are included in the article/Supplementary Material, further inquiries can be directed to the corresponding author.
