# Supplementary material for: Genome-wide Identification and Analysis of Splicing QTLs in Multiple Sclerosis by RNA-Seq Data
Source: Front Genet. 2021 Nov 12;12:769804. doi: 10.3389/fgene.2021.769804 (PMC8633104; doi:10.3389/fgene.2021.769804)
Supplement: Supplementary file 4 [file DataSheet1.docx]

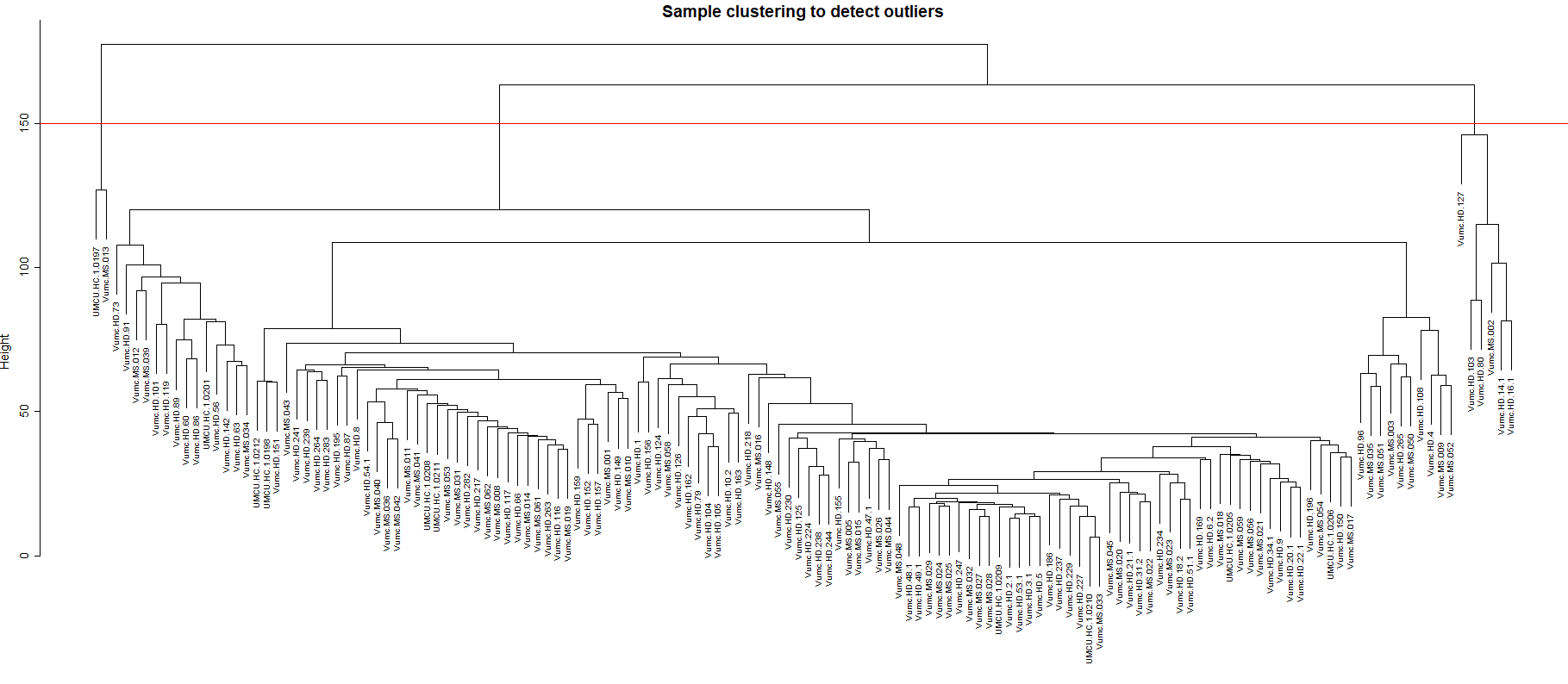


**Figure S1** Sample clustering to detect the outliers. The sample clustering is based on the gene expression profiles in each individual. It shows that there are a total of eight outlier samples according to the criterion (minimum cluster size$=10$ and cutting height$=1.5\times{10}^{2}$), and therefore they are removed for the following analysis.


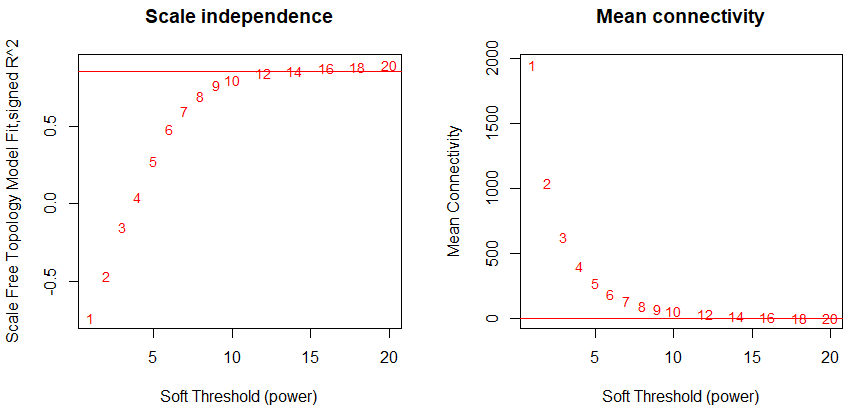


**Figure S2** Determination of satisfactory soft threshold power by network topology analysis. In the left panel, the soft threshold power equals 12 when the model fitting index R-squared reaches 0.85 for the first time. In the right panel, the soft threshold power still equals 12 when the mean connectivity approaches 0. Therefore, the optimum power value is set at 12 to ensure that the coexpression network follows scale-free topology criterion.
